# Effects of Wood Content and Modification on Properties of Wood Flour/Polybutylene Adipate Terephthalate Biocomposites

**DOI:** 10.3390/molecules28248057

**Published:** 2023-12-13

**Authors:** Wangwang Yu, Rui Qiu, Mengya Li, Wen Lei

**Affiliations:** 1 School of Mechanical Engineering, Nanjing Vocational University of Industry Technology, Nanjing 210023, China; 2 College of Science, Nanjing Forestry University, Nanjing 210037, China

**Keywords:** acetylation, biocomposite, polybutylene adipate terephthalate, property, wood flour

## Abstract

Biodegradable polymers have recently become attractive and have been increasingly used as matrix materials to replace fossil plastics due to concerns about the environmental issue. However, their application areas are limited due to their high costs and natural properties. In this study, we fabricated ecofriendly and economical polybutylene adipate terephthalate (PBAT) composites loaded with various concentrations of wood flour (WF) to investigate the effects on the PBAT and WF interfaces as well as the physical properties of the WF/PBAT biocomposites. Then, WF was acetylated with acetic anhydride, and the effect of WF acetylation on the mechanical and thermal properties of the biocomposites were investigated. The results showed that the tensile strength, tensile modulus, flexural strength and flexural modulus increased with WF loading in the composites, and acetylation could not only further increase these properties, but also increase the impact strength and elongation at break. The incorporation of WF would weaken the thermal stability of PBAT, but the thermal stability of the biocomposite could be improved after WF acetylation. The cold crystallization temperature and hydrophobicity of the WF/PBAT samples would be increased with the increasing load of the WF, while the melting enthalpy and the crystallinity of the samples reduced gradually. A morphological analysis of the modified composites revealed that the matrix exhibited greater interfacial interactions with the WF compared to the WF/PBAT. Considering the much lower cost of WF compared to PBAT, the improved properties of WF/PBAT biocomposites will make it economically competitive with other commercial polymers, and these biocomposites should have much wider application areas.

## 1. Introduction

Being entirely non-biodegradable, traditional plastics, such as polyethylene, polypropylene and polystyrene, have made the environment more seriously white polluted, and developing biodegradable polymer materials has become a research hotspot in recent years to reduce the impact of the applications of polymer materials on the environment.

Among commercially available biodegradable polymers, PBAT is an ideal candidate in the fields of agriculture, packaging films and medical devices [1]. As an aliphatic-aromatic random copolyester prepared by chemical synthesis from fossil resources [2], PBAT has high flexibility and excellent ductility, and it can be completely degraded within a few weeks by means of biological enzymes in the atmosphere. However, the high production cost and relatively low modulus and stiffness of PBAT restrict its wide commercial application. Complexing PBAT with natural fibers or their derivatives while maintaining its biodegradability and impact resistance has proven to be a practical method to solve these problems, and the obtained biocomposites displayed attractive application prospects for a range of single-use consumer goods, such as fast-food utensils, cosmetic containers and food containers [3]. For example, Yang et al. [4] modified ligninsulfonate nanoparticles using maleic anhydride (MLS) and then prepared MLS/PBAT composites by melting the blend. They found that the composite had increased tensile strength, elongation at break, tensile modulus and flexural modulus simultaneously than pure PBAT when 5% MLS was incorporated. Meanwhile, the apparent viscosity of PBAT was greatly decreased, and as a consequence, the processing properties of the blend system was improved. Jessica et al. [2] treated milled peach palm tree fibers known as “pupunha” with glycidoxypropyltrimethoxy silane (GPTMS), and prepared untreated and treated fiber-reinforced PBAT biocomposites. They found that the ultimate tensile strength and elongation at break decreased with the addition of both untreated and treated fibers. Nevertheless, the tensile moduli were significantly improved by the presence of the “pupunha” fiber, which also increased as the amount of the fiber in the composite increased. This effect was more pronounced for the systems containing GPTMS-modified fibers due to the improvement of the fiber–matrix interaction. The glass transition temperature increased, while the tan delta peak height in the dynamic mechanical properties testing curves decreased as the amount of fiber increased. Arvind et al. [3] dispersed hemp powder (HP) in PBAT to prepare HP/PBAT biocomposites using an extrusion process, using maleic anhydride-grafted PBAT (mPBAT) as a functional additive. They found that the mPBAT could improve the interfacial compatibility between the HP and PBAT; the tensile strength, toughness and impact resistance of the biocomposites were accordingly increased by around 209%, 300% and 90%; and the heat deflection temperature of the biocomposite containing 40% HP was about 60 °C greater than that of the neat PBAT.

As the by-product of the wood industry, wood flour (WF) is one of the abundant and renewable industry residues, it is cheap, light, biodegradable and easily available, and it also has a high strength-to-weight ratio; for these reasons, WF has been widely introduced into both thermosetting and thermoplastic polymers to form composites. The thermosetting polymers concerned included unsaturated polyester resin [5,6], epoxy resin [7,8], phenolic resin [9,10] and polyurethane [11,12]; the thermoplastic polymers included some traditional undegradable polymers, such as polypropylene [13,14], polystyrene [15,16], polyethylene [17,18], acrylonitrile butadiene styrene (ABS) [19,20] and polyvinyl chloride [21,22]; and some degradable polymers, such as polylactic acid (PLA) [23,24], polyhydroxyalkanoates (PHA) [25,26], polycaprolactone (PCL) [27,28] and poly(butylene succinate) (PBS) [29,30], were also included. The incorporation of WF had obvious effects on the properties of the polymers; however, no definite changing rules were suitable for all kinds of WF/polymer composites. Taking the tensile strength and modulus as examples, both the tensile strength and modulus increased when the WF content in the composites increased from 20 wt.% to 40 wt.% for the WF/PE composites [17], the WF content increased from 0 wt.% to 15 wt.% for WF/PCL [27], the tensile strength decreased, while the tensile modulus increased when the WF content increased from 0 wt.% to 30 wt.% for WF/PHA [25], and the WF content increased from 0 wt.% to 15 wt.% for WF/UP [6]. For the WF/PLA composites [23], the tensile strength increased when the WF content increased from 0 wt.% to 10 wt.% and then decreased; however, the tensile strength of the composite containing 20 wt.% of WF was almost the same as that of the composite containing 30 wt.% of WF, and the change in the tensile modulus was complicated, as the tensile moduli of the WF/PLA composites were 3.27 GPa, 3.63 GPa, 3.94 GPa, 3.84 GPa, 3.86 GPa and 3.00 GPa when the WF contents were 0 wt.%, 10 wt.%, 20 wt.%, 30 wt.%, 40 wt.% and 50 wt.%, respectively.

Regarding wood material-reinforced PBAT composites, more investigations have concentrated on the lignin/PBAT composites [4,31], and not so many studies have been reported on the WF/PBAT biocomposites. As aforementioned, WF is quite cheap, and its sales price in China is now about 800 CNY/ton (about 110 USD/ton), while that of PBAT is about 35,000 CNY/ton (about 4795 USD/ton), meaning the material cost will be reduced by 3420 CNY/ton (about 468.5 USD/ton) once 10 wt.% of PBAT is replaced by WF in the composite. The significantly reduced material cost will make the WF/PBAT biocomposite have strong market competitiveness.

For the above reasons, this paper focused on the investigation of the preparation and performances of WF/PBAT biocomposites, and emphasis was put on the investigation of the effects of the WF content and modification on the properties of the composites; the properties of neat PBAT was also investigated for comparison. The aim of this research was to improve the properties of PBAT while reducing its cost, and the ultimate destination is to promote the application of PBAT in more areas.

## 2. Results and Discussion

### 2.1. FTIR Analysis

**Figure 1:**
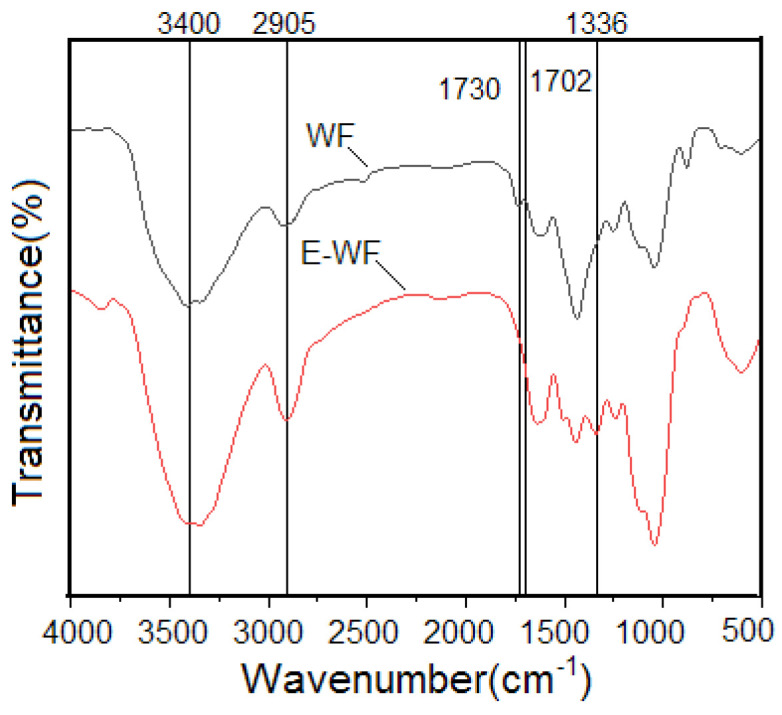
Fourier-transform infrared (FTIR) spectra of WF and E-WF.

The FTIR spectra of the WF and acetylated wood flour (E-WF) are presented in Figure 1. A comparison of the spectra of the WF and E-WF revealed several peaks in common. The wide bands around 3400 cm^−1^ for the WF and E-WF were due to the stretching vibration of the hydroxyl groups, and the peaks around 2905 cm^−1^ corresponded to the C-H stretching vibrations of aliphatic hydrocarbons [32].

The band around 1730 cm^−1^ in the spectrum of the WF was attributed to the carbonyl stretching vibrations of lignin and hemicellulose [33,34]; this absorbance disappeared in the spectra of E-WF, showing that the lignin and hemicellulose were removed during the treatment, which might be because of the alkaline treatment that was performed before the acetylation grafting reaction, as alkaline treatment was proved to be an effective method to remove the lignin and hemicellulose from WF [32,33]. After acetylation, two new absorbance peaks at 1702 cm^−1^ and 1336 cm^−1^ appeared, which might have resulted from the stretching vibration of the carbonyl and alkyl groups of acetic anhydride. There were no peaks at 1700 cm^−1^ and within the range from 1760 cm^−1^ to 1850 cm^−1^, indicating that the acetic anhydride and the by-product of acetic acid did not exist in E-WF. All of the results, as depicted above, confirmed that the WF was successfully acetylated through a chemical bonding process instead of only through physical absorption.

### 2.2. Effect of WF Content on Properties of WF/PBAT Biocomposites

#### 2.2.1. Visual Appearance

**Figure 2:**
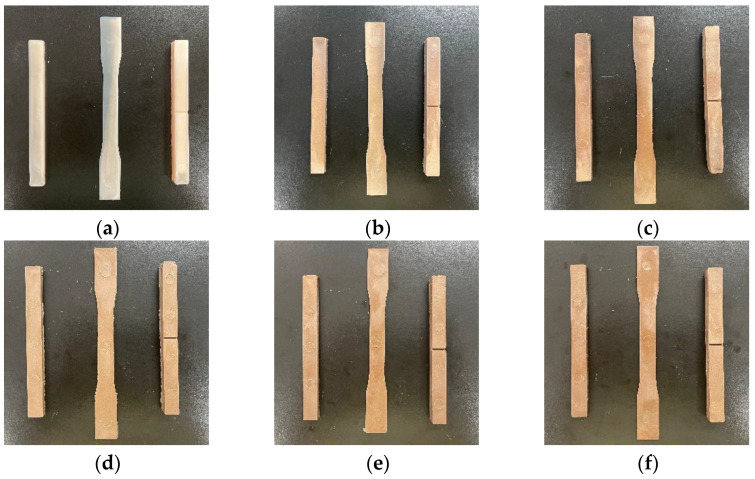
Visual appearance of neat PBAT and WF/PBAT biocomposites: (**a**) PBAT; (**b**) 10% WF/PBAT; (**c**) 20% WF/PBAT; (**d**) 30% WF/PBAT; (**e**) 40% WF/PBAT; (**f**) 50% WF/PBAT.

The photos of the injected samples were taken using a cell phone with a resolution of twelve million pixels, and they are illustrated in Figure 2.

From Figure 2, it could be seen that the injected PBAT samples were light yellow in appearance, the addition of the WF made the samples become more yellow and, generally, the changes in color were more obvious with the increasing dosage of WF in the composites, which was attributed to the yellow color of the WF itself.

#### 2.2.2. Mechanical Properties

**Figure 3:**
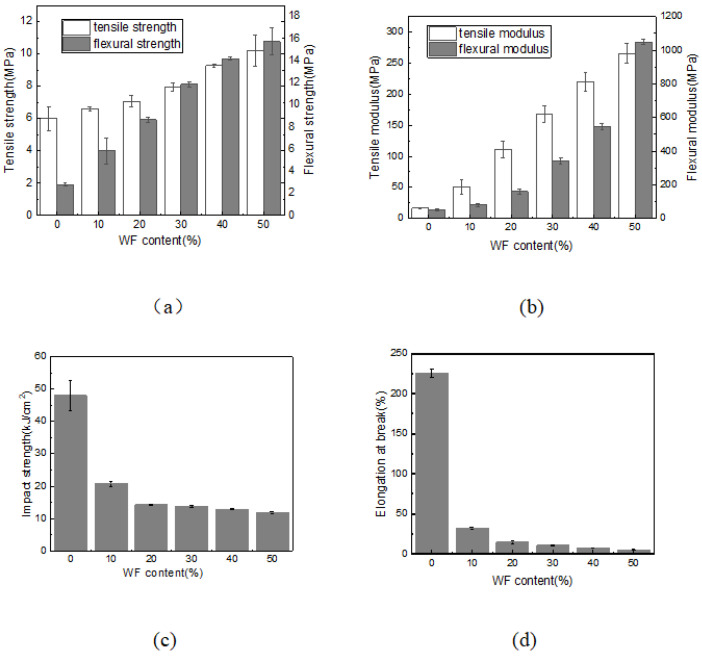
Mechanical properties of neat PBAT and WF/PBAT biocomposites: (**a**) tensile and flexural strengths; (**b**) tensile and flexural moduli; (**c**) impact strength; (**d**) elongation at break.

Several mechanical performances of the injected samples versus the WF content are illustrated in Figure 3.

From Figure 3a, the tensile strength of neat PBAT was 6.00 MPa, which fell into the range of the results obtained by Zhai et al. [35] and Mtibe et al. [36], and the flexural strength was 3.14 MPa, which was quite close to the 3.40 MPa value reported by Raksaksri et al. [37]. After complexing with WF, both the tensile and flexural strengths increased gradually with the content of WF, and the tensile and flexural strengths of the 50% WF/PBAT biocomposite were increased to 10.20 MPa by 70% and 15.72 MPa by 461.43% compared to those of the unfilled PBAT, respectively. The tensile and flexural moduli also showed similar changing trends. The increase in the tensile and flexural strengths and moduli showed the reinforcing effect of the WF [3], which may be due to the great strength and stiffness of cellulose and lignin in WF.

Besides these, Figure 3a also demonstrates that the absolute value of the tensile and flexural strengths of PBAT were very small, while the strengths could be increased greatly after the incorporation of WF; this enhancement would make the biocomposites suitable for applications in wider areas.

PBAT has a very good flexibility owing to its flexible groups such as ester groups, and its impact strength was as high as 48.00 kJ/m^2^, as illustrated in Figure 3c. When WF was introduced, however, the impact strength reduced greatly, and this reduction was the most obvious when 10 wt.% WF was used; in this situation, the impact strength was reduced by 56.71%. When 20 wt.% WF was used, the impact strength was reduced furthermore, and the impact strength only became 68.82% that of the 10% WF/PBAT. When more WF was used, however, the differences in the impact strengths of various samples turned to be much smaller, though they still decreased gradually.

PBAT has a good ductility by itself, and its elongation at break (EAB) was as great as 225.14% (Figure 3d); however, when WF was incorporated, the EAB decreased drastically, which is quite similar with the changing trend of the impact strength, where a higher WF content led to a smaller EAB, and the similar results have also been observed on HP/PBAT biocomposites, showing the relatively weak interaction between the natural fiber and PBAT chains [3].

#### 2.2.3. Thermal Stability

**Figure 4:**
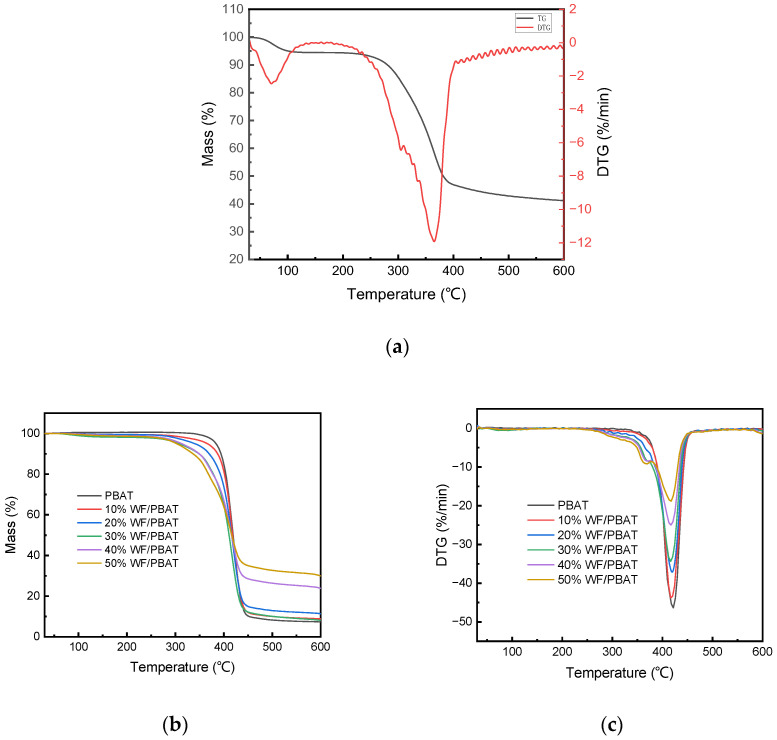
Thermogravimetric analysis of WF and injected samples under nitrogen atmosphere: (**a**) TG-DTG curves of WF; (**b**) TG curves of injected samples; (**c**) DTG curves of injected samples.

The TG-DTG curves of WF are presented in Figure 4a, and Figure 4b,c show the representative TG and DTG curves of the WF/PBAT biocomposites; the WF content in the biocomposites varied from 0 to 50 wt.%. As seen in Figure 4a, there were two obvious weight losses in the TG-DTG curves of the WF, and the obvious weight loss that occurred from room temperature to 100 °C was due to the evaporation of adsorbed moisture or small molecules in the WF. The degradation of hemicellulose in the WF should generally occur around 295 °C, while cellulose pyrolysis occurs at a higher temperature (315–400 °C) [38]; actually, the decomposition of cellulose and lignin occurred almost simultaneously in our study, as demonstrated in Figure 4a, and this phenomena was also observed by Hatakeyama et al. [39].

Similar with that of the WF, the thermogravimetric curves in Figure 4b and their derivative curves in Figure 4c show that all of the biocomposites lost a little mass when the temperature was raised from room temperature to 100 °C due to the evaporation of water or small molecules in the samples, but the main mass loss of each injected sample happened between 200 °C and 500 °C. The corresponding technical parameters in this temperature range are tabulated in Table 1.

The results showed that PBAT has a higher thermal stability due to the existence of terephthalate moieties of the polymer chain [40], and it began to decompose at 369.38 °C, which was much greater than that of the WF, as discussed above; as a result, the introduction of WF worsened the thermal stability of PBAT, and a greater proportion of WF in the biocomposite resulted in a poorer thermal stability of the samples. This could be evidenced from the gradually reduced Ti values of the samples listed in Table 1.

In addition, it could also be found that the decomposition of PBAT was realized in one step, the decomposition temperature range was from 369.38 °C to 450.02 °C and the peak temperature, T_p_, was 420.41 °C. A similar decomposition also happened for the 10% WF/PBAT and 20% WF/PBAT biocomposites, but when more WF was used, two peaks appeared in the DTG curves; one was near 420 °C due to the decomposition of PBAT, and the other was located around 370 °C, corresponding to the decomposition of WF, indicating that a higher dosage of WF would cause the phase separation between WF and PBAT.

It could thus be concluded that complexing PBAT with WF would worsen the thermal stability of PBAT, and a greater WF content would make the composites more thermal unstable.

#### 2.2.4. Melt and Crystallization Behavior

**Figure 5:**
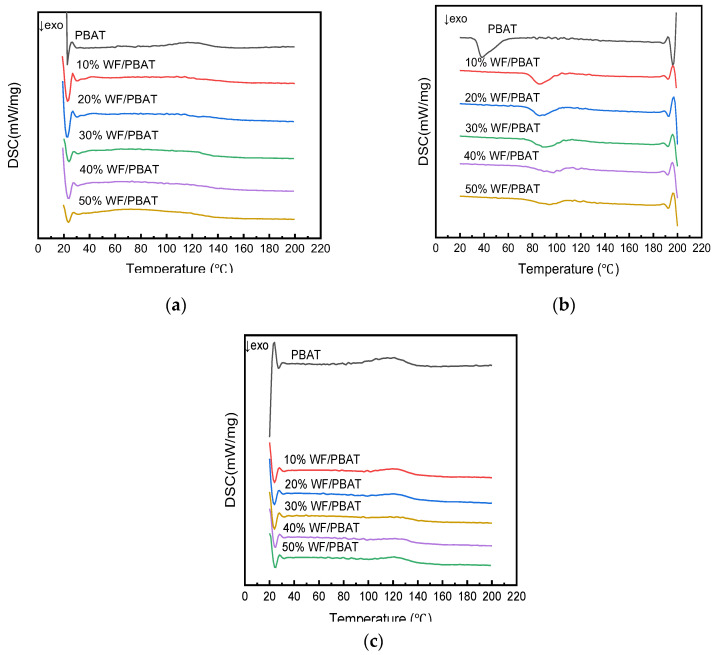
DSC thermograms of different WF/PBAT composites: (**a**) first heating; (**b**) cooling; (**c**) second heating.

In order to investigate the effect of the WF content on the melting and crystallization properties, a DSC analysis was carried out on the samples. The heating–run curves of the various WF/PBAT biocomposites obtained are shown in Figure 5. The total DSC characteristics of the WF/PBAT biocomposites are summarized in Table 2.

As can be seen from the DSC curves in Figure 5b, all of the samples showed only one crystallization peak during the cooling process, indicating that complexing with WF did not change the crystallization behavior of PBAT; however, the crystallization peak shifted to a higher temperature with the increase in the WF content in the composite. The calculated results of the DSC analysis in Table 2 revealed that with the increase in the WF content, the cold crystallization temperature rose monotonically, and the T_cc_ for neat PBAT was 71.3 °C, while that for the 50% WF/PBAT was 94.7 °C, which is 23.4 °C higher than that of neat PBAT, implying that WF could act as the heterogeneous nucleating agent in the composite [41,42]. When more WF was used, the crystallization peak became wider, showing that the solid particles of the WF prevented the diffusion of the molecular chain segments of PBAT to the nuclear agent, and the arrangement of the chain segments were accordingly limited; as a result, the crystallization rate became slow.

Figure 5c illustrates the second heating curves of the injected samples, which were a little similar than those in the first heating cycle (Figure 5a). The melting temperature of PBAT was 122.7 °C, which was very close to that of 124.3 °C reported by Jyoti Giri [43]; all the injected samples had similar melting peaks, and no obvious change occurred to their melting temperatures, but when more WF was used, the peak area became smaller, and the melting enthalpy reduced due to the dilution of the PBAT concentration with the incorporation of a higher loading of WF [40]. Consequently, the crystallinity of the samples was decreased, as evidenced in Table 2.

#### 2.2.5. Fracture Surface Morphology

**Figure 6:**
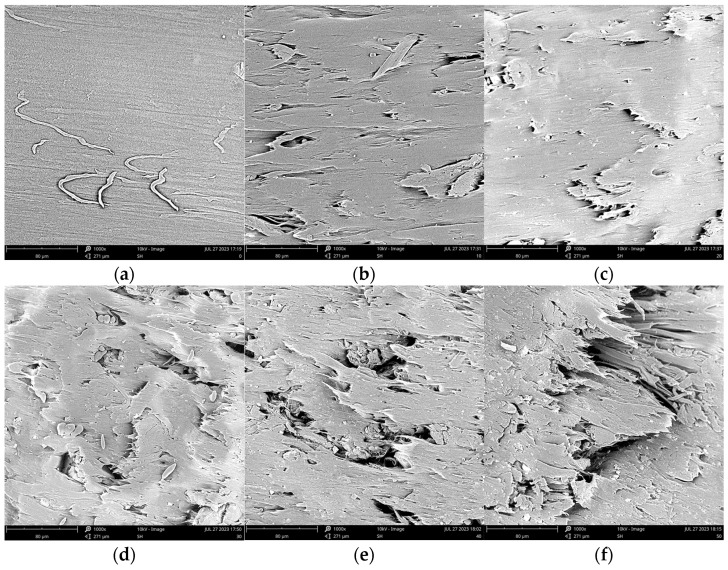
Scanning electron micrograph showing the fracture surface of the injected sample along its cross-section: (**a**) PBAT; (**b**) 10% WF/PBAT; (**c**) 20% WF/PBAT; (**d**) 30% WF/PBAT; (**e**) 40% WF/PBAT; (**f**) 50% WF/PBAT.

The microstructures of the injected samples were examined using a scanning electron microscope (SEM) with a magnification of 1000 times. The SEM micrographs from the fractured surfaces of the samples after the tensile test are presented in Figure 6.

The SEM micrographs revealed that the fracture surface of PBAT was homogeneous (Figure 6a), while the WF addition increased the morphology complexity. For the composites containing 10 wt.% and 20 wt.% WF, the fracture surfaces of the samples became a little rougher but were still generally uniform, the WF was wrapped with PBAT tightly and porosities or other defects could be found, as shown in Figure 6b,c. When more WF was added, on the one hand, the reinforcing effect became more obvious, and as a result, the tensile and flexural strengths and modulus became greater; on the other hand, some WF may gather together, and more defects, such as cracks, could be found in the SEM pictures. The pulling effect of wood flour led to a reduced elongation at break of the sample.

#### 2.2.6. Wettability

**Figure 7:**
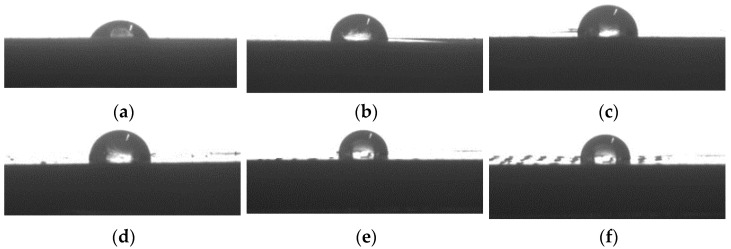
Photo of contact angle measurement: (**a**) PBAT; (**b**) 10% WF/PBAT; (**c**) 20% WF/PBAT; (**d**) 30% WF/PBAT; (**e**) 40% WF/PBAT; (**f**) 50% WF/PBAT.

The surface contact angle morphology of the injected samples are shown in Figure 7, and the results from the contact angle tests are presented in Table 3, corresponding to an average of three samples for each type of composite.

It was found that the surface wettability of the samples increased with the increasing dosage of WF, and the water contact angle for PBAT in Figure 7a and Table 3 was 59.9°; after being complexed with WF, the surface contact angle of the composites increased monotonically with the WF content, and the 50% WF/PBAT had the greatest contact angle of 95.2°, which was increased from that of the neat PBAT by 58.85%, indicating that the incorporation of WF was helpful for the enhancement of hydrophobicity of the samples, and could even change the composite from hydrophilicity to hydrophobicity. This phenomena was once found by Ayrilmis et al. [44] regarding WF/PLA composites; in their research, the contact angle of neat PLA was 67.8°, the WF/PLA composite containing 30% WF showed hydrophilicity, whose contact angle was 89°, while the composites containing 40% WF turned to be hydrophobic. In this composite, the contact angle became 97.3°.

### 2.3. Effect of WF Modification on Properties of WF/PBAT Biocomposites

#### 2.3.1. Mechanical Properties

The mechanical properties of the E-WF/PBAT biocomposites are listed in Table 4.

Comparing the data in Table 4 and the results for the 50% WF/PBAT biocomposites illustrated in Figure 3, it was clear that, after the modification on WF, the injected 50% E-WF/PBAT biocomposites had improved mechanical properties, and the tensile strength, tensile modulus, elongation at break, flexural strength, flexural modulus and impact strength values increased from those of the 50% WF/PBAT biocomposites by 27.35%, 15.07%, 156.94%, 12.60%, 73.21% and 26.80%, respectively. The improvement in the mechanical properties was due to the better interfacial adhesions in the composite [45]. The reasons should come from two aspects: on one hand, the acetylation removed waxy material from the fiber surface, and also removed lignin and hemicellulose in the WF; on the other hand, the acetylation made the hydrogen atoms of the hydroxyl groups be replaced by the acetyl group, and the surface polarity of WF was reduced. Consequently, the fiber–matrix interfacial bonding was enhanced, and the surface-free energy was increased, which were favorable for better properties of composites [46].

#### 2.3.2. Thermal Stability

**Figure 8:**
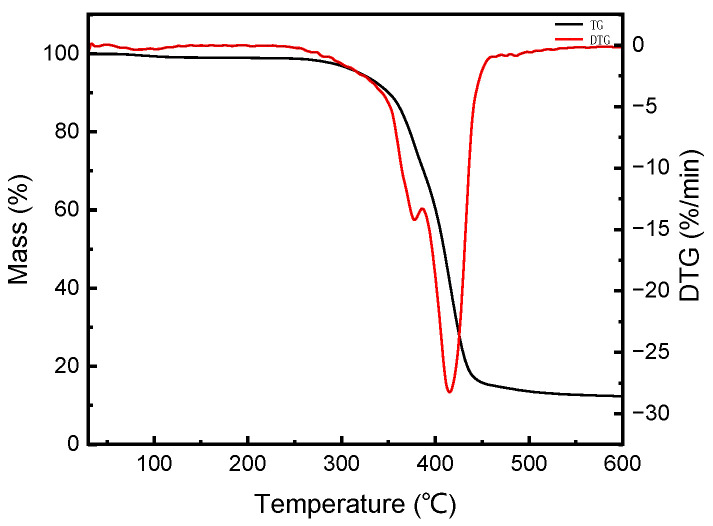
TG-DTG curves of E-WF/PBAT biocomposites.

The TG-DTG curves of 50% E-WF/PBAT biocomposites are shown in Figure 8.

From this picture, the T_i_, T_p,1_ and T_p,2_ values during the main thermal decomposition period (200–500 °C) were 351.79 °C, 378.51 °C and 415.07 °C, respectively. The greater T_i_ of 50% E-WF/PBAT than that of 50% WF/PBAT shown in Table 2 indicates that the thermal stability of the injected samples was enhanced after the WF modification; this enhancement was once observed for nonwoven unidirectional matted banana empty fruit bunch fiber/polypropylene composites by Zaman et al. [46]. In addition, the modified biocomposite had a greater T_p,1_ value and almost the same T_p,2_ value when compared with those of the unmodified biocomposite; the decreased difference between T_p,1_ and T_p,2_ of the E-WF/PBAT compared to that of the WF/PBAT revealed that the interfacial compatibility between the WF and PBAT was improved.

#### 2.3.3. Fracture Surface Morphology

**Figure 9:**
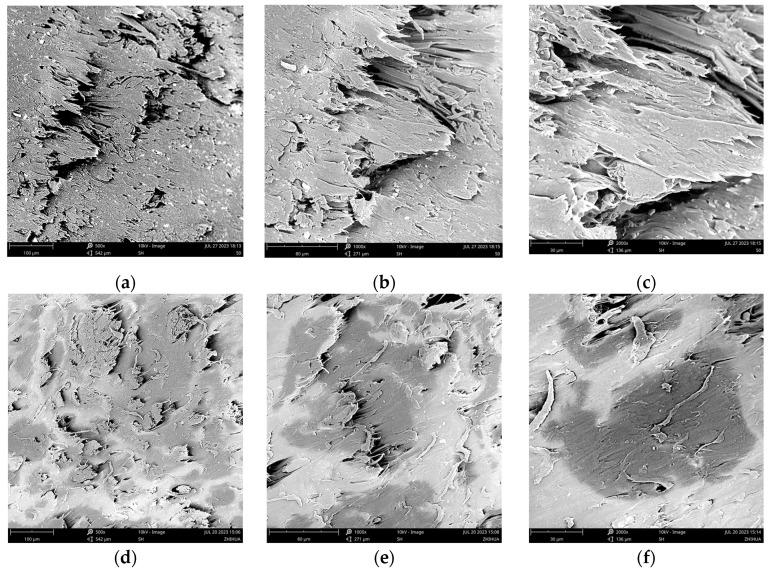
Cross-sectional morphologies of 50% WF/PBAT and 50%E-WF/PBAT at different magnifications: (**a**) 50% WF/PBAT, 500 times; (**b**) 50% WF/PBAT, 1000 times; (**c**) 50% WF/PBAT, 2000 times; (**d**) 50% E-WF/PBAT, 500 times; (**e**) 50% E-WF/PBAT, 1000 times; (**f**) 50% E-WF/PBAT, 2000 times.

For comparison, the fracture surface morphologies of the WF/PBAT and E-WF/PBAT biocomposites were observed under SEM at different magnifications, as shown in Figure 9.

For WF/PBAT, the fracture surface, as shown in Figure 9a–c, was heterogeneous; apparently, there existed some large porosities on the surface, and the fiber was not wrapped tightly by the matrix. For E-WF/PBAT, as illustrated in Figure 9d–f, however, the surface became much more homogeneous, all the fiber was wrapped by PBAT and no interfacial debonding could be observed, indicating that the acylation of WF improved the interfacial compatibility between the WF and PBAT, and this compatibility led to the improvement of both the mechanical properties and thermal stability of the biocomposites.

## 3. Experimental Procedure

### 3.1. Materials

PBAT in pellet forms was purchased from Xinjiang Blue Ridge Tunhe Sci. & Tech. Co., Ltd., China (Changji, China); WF, 80 mesh, was kindly supplied by Nanjing Dayuan Ecological Construction Group, China (Nanjing, China).

### 3.2. Modification of WF

#### 3.2.1. Alkaline Treatment

WF was dried at 105 °C for 24 h, a 5% sodium hydroxide solution was prepared by mixing sodium hydroxide and distilled water (the mass ratio of WF to the sodium hydroxide solution was 1:20), and then the dried WF was immersed in the hydroxide solution and stirred homogeneously; after 8h, the WF was taken out from the solution and washed until the eluent was neutral. After that, WF was dried at 105 °C to constant mass, and the alkaline-treated WF was obtained, named A-WF.

#### 3.2.2. Acetylation

A 5 wt.% acetic anhydride solution was prepared by mixing acetic anhydride and distilled water, and then A-WF was immersed in the acetic anhydride solution (the mass ratio of A-WF to the acetic anhydride solution was 1:20), and the solution with A-WF was kept at 120 °C for 1.5 h; next, the wood flour was taken out from the solution and washed until the eluent was neutral. Finally, the wood flour was dried at 105 °C to constant mass, and the acetylated WF was obtained, named E-WF.

### 3.3. Sample Preparation

Prior to blending, PBAT and WF were treated at 105 °C to constant masses to remove any traces of moisture. Then, the dried PBAT and WF were mixed for 15 min in different weight ratios, as presented in Table 5. Subsequently, the WF/PBAT mixture was extruded and pelletized using a twin-screw extruder machine (SHJ-20, Nanjing Giant Machinery Co., Ltd., Nanjing, China). The extruder temperatures from the hopper to the die were as follows: 105 °C, 110 °C, 110 °C, 110 °C, 110 °C and 105 °C. Finally, the pellets were injection-molded using an electric injection molding machine (CWI-90BV, Shanghai Jiwei Machinery Industry Co., Ltd., Shanghai, China) to obtain the samples for testing, and the injection temperature was controlled in the range from 110 to 120 °C.

Using the same steps, the composites containing E-WF were prepared, and the sample codes of the obtained composites were defined as 50% E-WF/PBAT.

### 3.4. Characterization and Determination of Properties

#### 3.4.1. FTIR Analysis

Fourier-transform infrared (FTIR) spectroscopy of WF was performed using Bruker attenuated total reflection–FTIR spectrometer (VERTEX 70, Bruker Optics, Ettlingen, Germany). IR spectrum was recorded in the range from 400 cm^−1^ to 4000 cm^−1^ with a resolution of 4 cm^−1^ at 32 scans/min. Before performing, WF was mixed with potassium bromide with the mass ratio of 1:100, and then compressed into tablets.

#### 3.4.2. Mechanical Strength and Modulus Testing

The tensile and flexural tests were performed in air at room temperature on a universal mechanical testing machine (E44.304, MTS Industrial Systems (China) Co., Ltd., Shenzhen, China) and a load frame with a 20 kN load cell, and a cross-head speed of 10 mm/min was used. The tensile test was performed in accordance with ASTM D 638-2010, and the tensile strength, tensile modulus and elongation at break were measured. The flexural test was performed in accordance with ASTM D 790-2010 using the same testing machine at a cross-head speed of 5 mm/min, and the flexural strength and flexural modulus were determined. Impact testing was carried out at room temperature with a pendulum electronic impact testing machine (XJC-25D, Chengde Precision Testing Machine Co., Ltd., Chengde, China). The test was carried out according to Chinese National Standard GB/T 1043.1-2008 [47].

#### 3.4.3. Thermal Stability Assessment

TGA was carried out employing a thermo-gravimetric analyzer (TG209F1, NETZSCH-Gerätebau GmbH, Selb, Germany) under nitrogen atmosphere. The samples (3–5 mg) were heated from 30 °C to 600 °C using a heating rate of 20 K/min. The initial thermal decomposition temperature (T_i_), the peak temperature at which the specimen decomposed the fastest (T_p_) and the terminal decomposition temperature (T_f_) were determined to distinguish differences arising from WF content and modification.

#### 3.4.4. Melt and Crystallization Behavior

Differential scanning calorimetry (DSC) analysis was carried out with 3–5 mg samples using a DSC instrument (DSC214, NETZSCH-Gerätebau GmbH, Germany). Samples were run using a heat/cool/heat cycle at a heating rate of 10 K/min from 20 °C to 220 °C under a nitrogen atmosphere. Samples were initially heated from 20 °C to 200 °C and held isothermally for 5 min to eliminate thermal history, residual moisture, and voids. Then, the sample was cooled down to room temperature and reheated to 220 °C. The transition temperatures and heat capacities were calculated via the NETZSCH analysis software(Proteus70). Equation (1) was used to calculate the crystallinity (χ) of PBAT:

(1) $$x_{c}=\frac{{|\Delta H}_{m}+{\Delta H}_{cc}|}{\omega \mathrm{\Delta H}*}$$

where x_c_ represents the degree of crystallinity of the sample, ω is the weight fraction of PBAT matrix in the sample, ΔH_m_ is the melting enthalpy change (J/g), ΔH_cc_ is the enthalpy change of cold crystallization (J/g) and ΔH* is the melting enthalpy of 100% crystalline PBAT (114 J/g [31]).

#### 3.4.5. Morphological Characterization

The morphology of the fractured surface of the specimen was observed by using a field-emission scanning electron microscope (SEM) (Hitachi SU 8010, Hitachi Corporation, Tokyo, Japan) at an accelerating voltage of 3 kV. For a better resolution, a thin layer of gold was sprayed on the surfaces of the samples before the SEM observation.

#### 3.4.6. Wettability Testing

The wettability of the injected samples was estimated with a contact angle system (DSA100; KRÜSS GmbH, Borsteler Chaussee, Germany) at room temperature. A 5 µL droplet of distilled water was dropped onto the surface and kept for 15 s, and then the contact angles from the images were measured at different points.

## 4. Conclusions

The following conclusions can be drawn from the experimental results of this study:

(1) For the mechanical measurements, the tensile strength, tensile modulus, flexural strength and flexural modulus of the WF/PBAT biocomposites increased with the WF loading, while the elongation at break and impact strength decreased. The morphological observation supported the test results of the mechanical properties.
(2) The incorporation of WF weakened the thermal stability of PBAT, and a greater WF loading led to a worse thermal stability of the biocomposites.
(3) The DSC studies revealed an increase in the cold crystallization temperature of the neat PBAT with the incorporation of WF, but the melting enthalpy and the crystallinity of the samples were reduced.
(4) The contact angle of distilled water on the surface of the sample increased gradually with the increasing content of WF, and the sample even turned from hydrophilic to hydrophobic when more WF was used.
(5) The acylated fiber composites showed increased mechanical properties and thermal stability, and the acetylation improved the interfacial bonding between the WF and PBAT, which was supported by the morphological observation.

To sum up, after complexing with WF, the cost of PBAT would be reduced significantly, and the properties of the resin would be changed. After WF acetylation, the mechanical properties and thermal stability of WF/PBAT biocomposites could be enhanced remarkably, which makes it possible for WF/PBAT biocomposites to be used in more areas.

## Figures and Tables

**Table 1:** Thermogravimetric analysis of samples with different proportions.

Sample Code	T_i_	T_p,1_	T_p,2_	T_f_
PBAT	369.38		420.41	450.02
10%WF/PBAT	361.22		417.14	448.62
20%WF/PBAT	346.94		418.36	446.32
30%WF/PBAT	344.90	370.12	415.51	444.65
40%WF/PBAT	342.86	369.80	415.10	443.57
50%WF/PBAT	342.04	368.96	415.14	440.82

**Table 2:** Thermal properties of PBAT and WF/PBAT biocomposites as calculated from the normalized DSC data.

Sample Code	T_cc_/°C	ΔH_c_ (J/g)	T_m_/°C	ΔH_m_ (J/g)	X_c_/%
PBAT	71.3	−17.39	122.7	13.43	11.78
10%WF/PBAT	85.9	−11.93	120.2	11.11	10.83
20%WF/PBAT	86.2	−9.32	120.9	9.44	10.35
30%WF/PBAT	88.4	−9.23	120.6	8.59	10.76
40%WF/PBAT	94.2	−6.73	123.9	7.15	10.46
50%WF/PBAT	94.7	−6.19	123.0	5.15	9.04

**Table 3:** Contact angles of water on the surfaces of injected samples with different contents of WF.

Sample Code	PBAT	10%WF/PBAT	20%WF/PBAT	30%WF/PBAT	40%WF/PBAT	50%WF/PBAT
Contact angle/°	59.9 ±6.0	82.8 ±7.5	88.0 ±1.8	90.1 ±4.0	92.1 ±3.9	95.2 ±4.2

**Table 4:** Mechanical properties of E-WF/PBAT biocomposites.

Sample Code	Tensile Strength (MPa)	Tensile Modulus (Mpa)	Elongation at Break (%)	Flexural Strength (Mpa)	Flexural Modulus (Mpa)	Impact Strength (kJ/m^2^)
50%E-WF/ PBAT	12.99 ±0.50	305.82 ±18.53	12.41 ±1.24	17.70 ±0.55	605.30 ±16.82	14.95 ±1.13

**Table 5:** Compositions of the composites with different WF loadings.

Sample Codes	PBAT	10%WF/PBAT	20%WF/PBAT	30%WF/PBAT	40%WF/PBAT	50%WF/PBAT
PBAT/wt.%	100	90	80	70	60	50
WF/wt.%	0	10	20	30	40	50

## Data Availability

Data are contained within the article.

## References

[1] Wang H., Wang B., Yuan T., Zheng L., Shi Q., Wang S., Song G., Sun R. (2020). Tunable, UV-shielding and biodegradablecomposites based on well-characterized lignins and poly(butylene adipate-co-terephthalate). Green Chem..

[2] Jessica S., Pereira S., Juliana M.F.S., Bluma G.S., Sebastien L. (2017). Fully biodegradable composites based on poly(butylene adipate-coterephthalate)/peach palm trees fiber. Compos. Part B.

[3] Arvind G., Bansri C., Boon P.C., Tizazu M. (2021). Robust and sustainable PBAT-Hemp residue biocomposites: Reactive extrusion compatibilization and fabrication. Compos. Sci. Technol..

[4] Yang X., Zhong S. (2020). Properties of maleic anhydride-modified lignin nanoparticles/polybutylene-co-terephthalate composites. J. Appl. Polym. Sci..

[5] Przemysław P., Andrzej P., Malgorzata M., Grazyna G., Barbara G. (2021). Synthesis, Characterization and testing of antimicrobial activity of composites of unsaturated polyester resins with wood flour and silver nanoparticles. Materials.

[6] Rahman M.R., Hamdan S., Hasan M., Baini R., ASalleh A. (2015). Physical, mechanical, and thermal properties of wood flour reinforced maleic anhydride grafted uyUnsaturated polyester(UP) biocomposites. Bioresources.

[7] Dwivedi U.K., Chand N. (2008). Influence of wood flour loading on tribological behavior of epoxy composites. Polym. Compos..

[8] Anna S., Piotr C. (2023). Modifification of epoxy compositions by the application of various fillers of natural origin. Materials.

[9] Chen H., Bahmani M., Humar M., Cheng D. (2020). Properties of wood ceramics prepared from thermo-modified Poplar. Forests.

[10] Guo X., Gao Q., Du D., Sun C. (2021). Effects of filling rate and resin concentration on pore characteristics and pProperties of carbon based wood ceramics. Materials.

[11] Kerim K., Yasin K., Ümit T. (2018). Mechanical, thermo-mechanical and water uptake performance of wood flour filled polyurethane elastomer eco-composites: Influence of surface treatment of wood flour. Holzforschung.

[12] Bi H.J., Ren Z.C., Guo R., Xu M., Song Y.M. (2018). Fabrication of flexible wood flour/thermoplastic polyurethane elastomer composites using fused deposition molding. Ind. Crops Prod..

[13] Klementina P.Č., Lidija F.Z., Lidija S.P., Marko B. (2020). Effect of wood fiber loading on the chemical and thermo-rheological properties of unrecycled and recycled wood-polymer composites. Appl. Sci..

[14] Slim S., Ferdaous L., Ahmed E., Anne B. (2022). Properties of wood polymer composites based on polypropylene/olive wood four: Efects of fber treatment and compatibilizer. Iran. Polym. J..

[15] Poletto M., Dettenborn J., Zeni M., Zattera A.J. (2011). Characterization of composites based on expanded polystyrene wastes and wood flour. Waste Manag..

[16] Kaseem M., Hamad K., Deri F., Ko Y.G. (2017). Effect of wood fibers on the rheological and mechanical properties of polystyrene/wood composites. J. Wood Chem. Technol..

[17] Aina K.S., Oladimeji A.O., Agboola F.Z., Oguntayo D.O. (2022). Dimensional stability and mechanical properties of extruded-compression biopolymer composites made from selected Nigerian grown wood species at varying Proportions. Sci. Rep..

[18] Patti A., Cicala G., Acierno S. (2021). Rotational Rheology of Wood Flour Composites Based on Recycled Polyethylene. Polymers.

[19] Phung A.T., Dzung H.T., Linh N.P.D., Duc V.M., Liem N.T. (2023). Acrylonitrile butadiene styrene/wood sawdust particles composites: Mechanical and morphological properties. Iran. Polym. J..

[20] Threepopnatkul P., Teppinta W., Sombatsompop N. (2011). Effect of Co-monomer Ratio in ABS and Wood Content on Processing and Properties in Wood/ABS Composites. Fibers Polym..

[21] Zong G.G., Hao X.L., Hao J.X., Tang W., Fang Y.Q., Ou R.X., Wang Q.W. (2020). High-strength, lightweight, co-extruded wood flour-polyvinyl chloride/lumber composites: Effects of wood content in shell layer on mechanical properties, creep resistance, and dimensional stability. J. Clean. Prod..

[22] Li J.L., Huo R.L., Liu W.Y., Fang H., Jiang L., Zhou D. (2022). Mechanical properties of PVC-based wood–plastic composites effected by Temperature. Front. Mater..

[23] Mirko K., Milan S., Murčo O., Manja K.K. (2018). Effect of wood content in FDM filament on properties of 3D printed parts. Mater. Today Commun..

[24] Jubinville D., Tzoganakis C., Mekonnen T.H. (2022). Recycled PLA-Wood flour based biocomposites: Effect of wood flour surface modification, PLA recycling, and maleation. Constr. Build. Mater..

[25] Musioł M., Jurczyk S., Sobota M., Klim M., Sikorska W., Zięba M., Janeczek H., Rydz J., Kurcok P., Johnston B. (2020). (Bio)Degradable Polymeric Materials for Sustainable Future-Part 3: Degradation Studies of the PHA/Wood Flour-Based Composites and Preliminary Tests of Antimicrobial Activity. Materials.

[26] Tian J., Zhang R., Wu Y.H., Xue P. (2021). Additive manufacturing of wood flour/polyhydroxyalkanoates (PHA) fully bio-based composites based on micro-screw extrusion system. Mater. Des..

[27] Cintra C.S., Braga N.F., Morgado G.F.D., Montanheiro T.L.D., Marini J., Passador F.R., Montagna L.S. (2022). Development of new biodegradable composites materials from polycaprolactone and wood flour. Wood Mater. Sci. Eng..

[28] Wu C.-S. (2004). Analysis of Mechanical, Thermal, and Morphological Behavior of Polycaprolactone/Wood Flour Blends. J. Appl. Polym. Sci..

[29] Weng F.Q., Liu X.M., Koranteng E., Ma N., Wu Z.S., Wu Q.X. (2019). Structure and properties of a compatible wood-plastic composite prepared by using poly(butylene succinate)-based polyurethane prepolymer. Polym. Compos..

[30] Park C.-W., Youe W.-J., Han S.-Y., Park J.-S., Lee E.-A., Park J.-Y., Kwon G.-J., Kim S.-J., Lee S.-H. (2019). Influence of Lignin and Polymeric Diphenylmethane Diisocyante Addition on the Properties of Poly(butylene succinate)/Wood Flour Composite. Polymers.

[31] Botta L., Titone V., Teresi R., Scarlata M.C., Re G.L., Mantia F.P.L., Lopresti F. (2022). Biocomposite PBAT/lignin blown films with enhanced photo-stability. Int. J. Biol. Macromol..

[32] Teklu T., Wangatia L.M., Alemayehu E. (2017). Effect of Surface Modification of Sisal Fibers on Water Absorption and Mechanical Properties of Polyaniline Composite. Polym. Compos..

[33] Wu J., Yi Z.X., Zhong T., Zhang W., Chen H. (2023). Bamboo slivers with high strength and toughness prepared by alkali treatment at a proper temperature. J. Wood Sci..

[34] Thygesen A., Fernando D., Ståhl K., Daniel G., Mensah M., Meyer A.S. (2021). Cell wall configuration and ultrastructure of cellulose crystals in green seaweeds. Cellulose.

[35] Zhai X.S., Wang W.T., Zhang H., Dai Y.Y., Dong H.Z., Hou H.X. (2020). Effects of high starch content on the physicochemical properties of starch/PBAT nanocomposite films prepared by extrusion blowing. Carbohydr. Polym..

[36] Mtibe A., Hlekelele L., Kleyi P.E., Muniyasamy S., Nomadolo N.E., Ofosu O., Ojijo V., John M.J. (2022). Fabrication of a Polybutylene Succinate (PBS)/PolybutyleneAdipate-Co-Terephthalate (PBAT)-Based Hybrid SystemReinforced with Lignin and Zinc Nanoparticles for PotentialBiomedical Applications. Polymers.

[37] Raksaksri L., Ruksakulpiwat Y., Udomkitpanya T. (2023). The Properties of Tannery Waste Addition as a Filler Based on Two Types of Polymer Matrices: Poly(Butylene Adipate-Co-Terephthalate) (PBAT) and Poly(Butylene Succinate) (PBS). Adv. Polym. Technol..

[38] Quitadamo A., Massardier V., Valente M. (2019). Eco-Friendly Approach and Potential Biodegradable Polymer Matrix for WPC Composite Materials in Outdoor Application. Int. J. Polym. Sci..

[39] Hatakeyama H., Ohsuga T., Hatakeyama T. (2014). Thermogravimetry on wood powder-filled polyurethane composites derived from lignin. J. Therm. Anal. Calorim..

[40] Lule Z.C., Kim J. (2021). Properties of economical and eco-friendly polybutylene adipate terephthalate composites loaded with surface treated coffee husk. Compos. Part A.

[41] Deng Y., Thomas N.L. (2015). Blending poly(butylene succinate) with poly(lactic acid): Ductility and phase inversion effects. Eur. Polym. J..

[42] Dai X., Cao Y., Shi X.W., Wang X.L. (2016). Non-isothermal crystallization kinetics, thermal degradation behavior and mechanical properties of poly(lactic acid)/MOF composites prepared by melt-blending methods. RSC Adv..

[43] Giri J., Lach R., Le H.H., Grellmann W., Saiter J.M., Henning S., Radusch H.J. (2020). Structural, thermal and mechanical properties of composites of poly(butylene adipate-co-terephthalate) with wheat straw microcrystalline cellulose. Polym. Bull..

[44] Ayrilmis N., Kariž M., Kuzman M.K. (2019). Effect of wood flour content on surface properties of 3D printed materials produced from wood flour/PLA filament. Int. J. Polym. Anal. Charact..

[45] Preampree S., Thanyapanich T., Boonmahittsud A., Intatha U., Tawichai N., Soykeabkaew N. (2020). Effects of mold sealing and fiber volume fraction on properties of rice straw/unsaturated polyester biocomposites. ScienceAsia.

[46] Zaman H.U., Khan R.A. (2021). Acetylation used for natural fiber/polymer composites. J. Thermoplast. Compos. Mater..

[47] (2008). Plastics—Determination of Charpy Impact Properties—Part 1: Non-Instrumented Impact Test. https://www.chinesestandard.net/PDF/English.aspx/GBT1043.1-2008.

